# Foreign Correspondence

**Published:** 1857-05

**Authors:** W. F. Westmoreland

**Affiliations:** Professor of the Principles and Practice of Surgery in the Atlanta Medical College


### Medical and Surgical Journal.

Vol. II.] MAY, 1857. [No. 9

### ORIGINAL COMMUNICATIONS.

### ARTICLE I.

### FOREIGN CORRESPONDENCE.

By W. F. Westmoreland, M. D., Professor of the Principles and Practice of Surgery in the Atlanta Medical College.

Paris, February 10th, 1857.

*Dear Doctor*:—I visited, this morning, the wards of M. Chassaignac at La Riboisiéne, and, although I had a fatigueing morning's walk--the hospital being situated in the extreme northern part of the City, some four miles from the School of Medicine,—I was fully repaid for my trouble.

M. Chassaignac is the inventor of the *écraseur*, an instrument alluded to in a previous letter. Upon this instrument, its application, and the advantages of the dry incision, or the incision without hemorrhage, (*incision séche*,) he has recently published a work of 550 pages. The *écraseur* which he uses in almost every operation, when it is possible to circumscribe the lesion with the noose of the chain, consists in a chain with links similar to the chain saw; in fact, is the chain-saw without the teeth; the ends of the chain are passed through a stout bar, accurately filling the openings made for their passage, and attached by means of hooks to a smaller bar which slides in the greater. The small bar to which the chain is attached, is moved by means of a screw, so that the operation, after adjusting the instrument, may make constriction upon any substance placed in the noose of the chain, and to any desirable extent, by merely turning a screw. It will be readily seen that this instrument acts upon the principal of the original *serre-nœud,* it being, however, much stronger, and more conveniently managed—dividing the tissues by constriction; but instead of a few hours or days, as by the *serre-nœud,* the section is accomplished in a few minutes, and without the loss of more than a few drops of blood, however vascular the part.

The principal lesions in which he uses the *écraseur* are: hemorrhoidal tumors, prolapsus of the rectum, polypus of the rectum and uterus, all lesions requiring a partial or complete amputation of tongue, fistula in ano, cancer of the rectum, cancer of the neck of the uterus, varicocele, sarcocele, phymosis, lesions requiring amputation of the penis, sub-cutaneous tumors as lipoma, erectile tumors, naso-pharingeal polypus, &c., &c. Substituting for the bistoury, as will be seen in quite the majority of operations, his favorite instrument.

He performed, this morning, three operations with the *écraseur,* for hemorrhoidal tumor, cancer of the umbilicus, and varicocele, and I am forced to acknowledge, that in each the operation was very satisfactory. The hemorrhoid tumor was small and external; it was removed in less time than two minutes without the loss of a drop of blood. M. Chassaignac assured me that it was the usual result of the operation, even when the tumor was interu and of large size, the only difference, the large tumors requiring more time. The umbilical cancer, which I should have had many scruples in attacking with the bistoury, he transfixed by passing pins between its hardened base and the peritoneum, so that the instrument would not come in contact with this membrane, passing the chain of the ecraseur above the pins. The extirpation of the tumor was accomplished in about ten minutes, and without the loss of but a few drops of blood. It would appear, without reflection, that after the section there would appear a large gaping wound the size of the base of the tumor, which was not the case. Before the tissues are divided, there is always a pedicle or neck formed, which exists after the section, the approximation of the divided tissues being so perfect, in quite a number of cases, as to prevent the contact of air with the wound, thus approaching the subcutaneous section. Pins were passed through the constricted neck and secured by a ligature to prevent the possibility of the retraction of the skin.

The varicocele was, as is usual, upon the left side, the veins being of large size. The operation was as follows: He first passed three pins behind the veins, as in the ordinary operation by the pins and ligature, the pins being from a third to half an inch apart, he next passed a ligature around the three pins, not as in the operation above alluded to, but embracing the three pins at the same time, the ligature was tightened forming a neck or pedicle behind the pins, thus separating that portion of the varicose veins embraced by the ligature from the artery and spermatic cord. The chain of the ecraseur was now placed around the neck thus formed, and the whole extirpated. From an inch to an inch and a half of the veins were excised. No hemorrhage. The skin was allowed to retract so that the point occupied by the veins would fill up by granulations, forming a cicatrix which would effectually prevent their reproduction.

M. Chassaignac has performed this operation several times with success. I examined, in his wards, this morning, a case upon which he had performed the same operation two weeks ago. The wound is granulating beautifully. No accidents had occurred.

During the clinic he presented several patients upon whom he had operated with the ecraseur for different diseases, and all with satisfactory results.

There are several recent improvements upon the original instrument, one in which wire is used instead of the chain, others differing only in the form and application of force to the chain; I doubt very much, however, whether any one of them have any claims over M. Chassaignac's instrument. It is more than probable, that, like every thing else, when first introduced either in medicine or surgery, too much is claimed for this instrument—that it is often used in cases where it is not applicable, or at least where the bistoury would give better results. It is, however, a most valuable addition to our list of surgical implements, and in several diseases, has superior claims to any other method of operating.

M. Chassaignac has introduced a new plan of treating abscesses, for which he claims as much or, perhaps, more than he does for the écraseur. This new method of treatment consists in a system of drainage by means of small India rubber tubes pierced by numerous holes. These tubes are passed through the abscess by means of a trocar and canula for the purpose, the number of tubes, depending upon the size of the abscess; after the canula is withdrawn the ends of the tubes are knotted to prevent their escape from the abscess, giving them the appearance of an ordinary seton. The pus, as fast as formed, enters the tube by means of the numerous holes by which it is pierced, and is discharged externally, thus obliterating the cavity by permitting the walls of the abscess to contract upon itself. He has no fears of the introduction of air and the decomposition of the pus, as in all his experiments he has never witnessed such an accident. He adopts this plan of treatment as well in the acute, as in abscesses by congestion. I saw in his wards, this morning, between fifteen and twenty cases under treatment—five or six were abscesses of the mammary gland. He passed, this morning, three tubes through an enormous abscess of the lumbar region symptomatic of Pott's disease of the spine. I learn that he is preparing a report to present to the *Société de Chirurgie* with the rise of three hundred cases treated by this method. He treats hydrocele in the same way. There are at present, in his wards, two cases with the tube above described, passed through the tunica vaginalis.

I examined a patient this morning, from whom he had amputated a leg with nitric acid. The operation was performed in November last. The wound has healed beautifully. No one, by an examination of the stump, would suspect the mode of amputation. It required five applications of the acid—the first two being excessively painful. The applications were made twice a day, making two days and a half. M. Chassaignac says that the acid may be applied every two hours, thus completing the amputation in less than twelve hours. It is evident that in America, where we amputate with such impunity, when death from amputation is an exception to the general result, we would never think, for a moment, of adopting a method so tedious, and so excessively painful to the patient. In Paris, however, it is different. Quite the majority of patients who submit to an amputation in the hospitals of this city, die in from three to ten days after the operation, of purulent infection. In the larger amputation, as of the thigh, &c., recovery is an exception to the general result. This fatality appears to be confined strictly to the city, as at an hospital at Versailles, only twelve miles of Paris, amputations are followed by the most favorable results. The same of the hospitals in the various provinces in France. I saw a patient die, a few days ago, in the wards of M. Nelaton, from an amputation of the index finger. In speaking of the death of this patient, M. Nelaton discussed to some extent the cause of this very fatal accident. He is of the opinion that there should be erected, near Paris, a hospital for the reception of patients requiring amputation—that amputation should not be performed in the city, except under peculiar circumstances. Surgeons, there are to some extent, excusable for experimenting, as the discovery of any method by which amputations could be performed without the risk of being followed by that terrible accident Pyema, would be one of the greatest additions to modern surgery, at least so far as the city of Paris is concerned.

Yours ever,

W. F. WESTMORELAND.

